# A response to “Comparative efficacy of LifeVac® and Heimlich Maneuver in simulated airway obstruction”

**DOI:** 10.1016/j.jped.2025.101427

**Published:** 2025-08-09

**Authors:** Simon Gould

**Affiliations:** Australian Resuscitation Advisory Network (ARAN), Newcastel, East Melbourne, New South Wales, Australia

I read with interest this recent article [1] and wish to provide some perspective in regard to some significant weaknesses in the methodology, conclusions, and narrative.

The methodology of the experiment assumed that an adult choking manikin is a proxy for pediatric anatomy. However, there are significant differences between pediatric anatomy e.g., airway shape; and also the anatomical and physiological behavior of a manikin with no living structures. The manikin used in the studies may have more “human-like features”, but it is not a perfect proxy, particularly for a pediatric. The authors claim that the manufacturer informed them this adult manikin “is considered suitable for simulating choking events in young children, because airway mechanics are similar regardless of size”, however, there is no evidence to support this assertion or that it translates in any way to live subjects of a different age.

While the study concludes that both measures have efficacy, it also distinguishes pressure differentials as significant. However, this observation has no relevance, as efficacy was similar with each method, with no demonstrated correlation between higher positive pressure gradients and efficacy, only a correlation between higher pressure gradients and injury i.e., an undesirable outcome.

Regarding first aid measures having little evidence, this is sadly true, and some e.g., ANZCOR- Chest Thrusts used instead of Heimlich Maneuver (called abdominal thrusts by convention) in Australia and New Zealand, have no evidence or rationale for their use [2]. However, the historical references to injuries in regard to specifically abdominal thrusts were addressed by ILCOR after a risk review that determined that injuries were predominately when performed on patients under 1 year and when users were not trained but had heard or read about the technique. ILCOR made appropriate changes to the recommendations so that the method is only recommended for patients over 1 year, and all CPR courses included the application of abdominal thrusts (except Australia). This resulted in the effective mitigation of injuries, and more recent data does not indicate this risk in relation to abdominal thrusts. What the study did not mention is that the finding of ILCOR showed evidence of significant injury with all first aid measures, but none related to LifeVac, https://costr.ilcor.org/document/removal-of-foreign-body-airway-obstruction-tfsr-costr

This fact was also overlooked by the ARC in Australia.

The article goes on to claim that literature on the LifeVac is “sparse”. However, this is a flawed assumption as there have been no <10 peer-reviewed studies in the last 5 years with *a* > 88 % acceptance of LifeVac® safety and efficacy from the data. The studies and usage data cited in this article were up to 2020 [3] i.e., a small and historically irrelevant sample that only reviewed studies with small sample sizes for LifeVac usage and cited studies that were corrected by authors in later research, including a 10-year case series [4]. One must ask, “sparse” compared to what? No first aid measure has strong evidence, nor an RCT (i.e., real-life studies) have or can ever be conducted on live subjects using choking measures or devices, as this is unethical and not a bar for the recommendation of poorly evidenced first aid measures. However, the underlying false equivalence being made in this, and other studies is that LifeVac® is designed to replace first aid measures; this is categorically untrue but is part of the false narrative used commonly used to raise the level of evidence necessary for acceptance.

One of the significant assumptions made by the study is that the ability to generate increased inter-abdominal and intrathoracic pressure is a constant when using the Heimlich Maneuver. Physiologically, this ability is reliant on the patient having a sufficient residual lung volume at the time of the choking and that this remains constant throughout the repeated application of first aid measures (remembering that the Heimlich Maneuver is only part of a suite of measures used in sequence). While this is true in a manikin, it is not for a live subject. Conversely, the negative external pressure generated by the LifeVac® is a constant, making it effective when first aid measures fail or are not feasible. The ability of first aid measures to generate positive pressure decreases the force required to generate sufficient positive pressure increases, and so does the risk of injury.

Although LifeVac® has documented all case reports (unlike first aid measures, where there is no reporting, surveillance, or investigation of failure/harm and success) the authors erroneously claim that data showing the ease of use of the LifeVac® cannot be true, and therefore there must be the need for “regular and systematic training”. There are several issues with this statement. Firstly, whilst regular training is helpful, there is no acknowledgment that the early injuries noted with abdominal thrusts were due to insufficient training because it has a higher risk of injury. Conversely, the risk profile for LifeVac® is low even in the hands of an untrained novice. There are studies that show ease of use [5,6]. Also given that the LifeVac® device is only indicated for use after failure of first aid measures, the only alternative is likely severe brain injury or death. These factors have all already been considered and managed by regulators.

The authors’ conclusions made in the discussion section of this study, despite the evidence, show a bias toward a historical narrative in the clinical/ academic community. The findings confirm the efficacy of the LifeVac®, but we are asked to interpret this with “caution”, but not the efficacy or safety of the Heimlich Maneuver. A truly comparative study would not make this biased statement. The need for “greater training” for LifeVac® but not Heimlich Maneuver (the source of the injuries) is called for based on the assumption of the authors but not from the evidence; again, reinforcing a historically biased narrative. The authors make the further assumption that the application of the device “may involve certain complexities”. Apart from no explanation of the meaning of this statement, it is likely this caveat is designed to support the historical narrative of a. skepticism of new technologies (regardless of the evidence [5,6]), and; b. that the use of any medical device (especially a mask) is a skill requiring clinical training to master. The major error in the conclusions still remains that the pressure gradient is the only determinant of efficacy. Back blows create a higher pressure for a shorter time interval than abdominal thrusts and thus are recommended as the primary step, as this is most effective on smooth objects high in the airway. Abdominal thrusts have a lower maximum pressure but with a longer time interval i.e., are better at shunting more tightly impacted obstructions. Similar studies using manikins [5] have shown significantly higher efficacy with LifeVac® over Heimlich Maneuver (97/71 % on first application) and no significant efficacy with the DeChoker® [7].

## Author contribution

All contributions by the lead author.

## Funding

Not applicable.

## Conflicts of interest

The author is the owner of LifeVac Australia.
